# Current landscape of clinical trials for HPV-positive head and neck squamous cell carcinoma (HNSCC)

**DOI:** 10.3332/ecancer.2022.1447

**Published:** 2022-09-20

**Authors:** Yara T Bteich, Jad E Hosri, Jad A Wehbi, Lea R Daou

**Affiliations:** Lebanese University, Faculty of Medical Sciences, Beirut, Lebanon; † Co-first authors; a https://orcid.org/0000-0001-5672-8920; b https://orcid.org/0000-0002-7781-3486; c https://orcid.org/0000-0001-7093-8203; d https://orcid.org/0000-0003-2486-0542

**Keywords:** HPV-positive, chemotherapy, chemoradiotherapy, head and neck squamous cell cancer, clinical trials

## Abstract

This study aims to determine the current state of clinical trials regarding HPV-positive head and neck squamous cell carcinoma (HNSCC). Clinical trials were filtered to fit the study’s aim using Clinicaltrials.gov: trials concerning HNSCC specifically those related to HPV done between January 2005 and December 2020 were extracted and information regarding location, duration, phases, patient recruitment, trial status, results, primary outcome, type of intervention and publication status were collected and analysed. As a result, 123 trials were included. North American countries (USA and Canada) conducted more than two-thirds of the trials (72.4%) compared to European countries and the rest of the world. Trials in phase II constituted more than half of those included in this study (53.7%). From the 123 trials included in this study, only 30 had their NCT identification number linked to publications, but less than half (46.7%) of the publications stemmed from trials with results. Drug combination was the most widely studied treatment modality. Despite falling in the middle of the spectrum with respect to the number of trials when compared to other diseases, our research highlights the need for even more trials tackling multiple aspects of HPV-positive HNSCC.

## Introduction

Head and neck squamous cell carcinoma (HNSCC) represents malignancies derived from the mucosal epithelium of the oral cavity, pharynx and larynx. HNSCC can occur following infection with oncogenic human papillomavirus (HPV) strains (called HPV-positive HNSCC) or following exposure to carcinogens mainly found in tobacco (called HPV-negative HNSCC) [1]. The prevalence of HPV-positive HNSCC varies depending on HPV vaccine availability and awareness, while that of HPV-negative HNSCC depends on patterns of tobacco and alcohol consumption. This is reflected by the high prevalence of oral cancer in South Central Asia and Melanesia [2] due to high consumption of betel nuts with or without tobacco [3, 4] and in Eastern and Western Europe [2] due to high tobacco consumption. HPV-positive HNSCC has a better prognosis as compared to other causes of HNSCC [1]. The mainstay of treatment of this subtype comprises surgical resection with or without adjuvant treatment by radiation or a combination of chemotherapy and radiation. Treatment choice and success depend on the cancer’s type and stage. For this reason, several trials were conducted to determine the most appropriate treatment type and regimen, but the output of such trials has not yet been evaluated. Available reviews in the literature focus on a single modality for the treatment of HNSCC, immunotherapy [5, 6]. Our study aims to conduct a review of clinical trials studying the treatment of HPV-positive HNSCC executed between 2005 and 2020 to assess their characteristics, outcomes and publication aggregate.

## Materials and methods

### Search strategy and selection criteria

**Figure 1.**
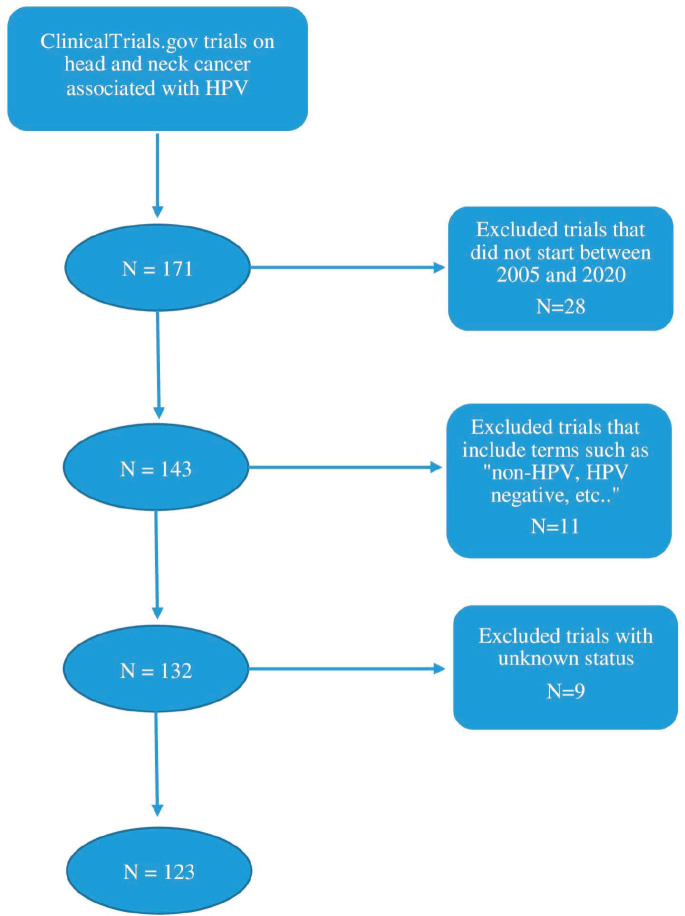
Schematic representation of the clinical trial selection process.

The authors retrieved all data and information on clinical trials from ClinicalTrials.gov. In the advanced search criteria, the condition or disease was chosen as “head and neck cancer” and “HPV” was the associated other term. Only interventional studies were considered as well as those starting between 01/01/2005 and 31/12/2020. This interval of time was chosen since more trials were conducted as of 2005. Therefore, to ensure the completion of a majority of the included trials, the year 2020 was chosen as the end of the time interval. Trials including terms such as “non-HPV, HPV-negative, HPV-unrelated” were excluded. Trials with unknown status were also excluded from this study. A total of 123 studies were included after a selection process based on inclusion and exclusion criteria as shown by the schema in Figure 1.

### Data collection

All information provided by the ClinicalTrials.gov registry was gathered. These include location, phase of the trial, number of patients enroled, trial status, duration, primary outcome, result status, type of intervention and NCT number.

Phases were classified as not applicable, I, I/II, II, II/III, III and IV. The term ‘not applicable’ is defined by the official clinical trials website as those without FDA-defined phases, including trials of devices or behavioural interventions [7]. The trial statuses that were considered include terminated, completed, recruiting, active but not recruiting, withdrawn and suspended. Trial duration was calculated as the difference between the trial start date and completion date. Primary endpoints/outcomes of clinical trials were defined in Table 1.

The authors divided the types of intervention into five main categories and two others: ‘Drug’ which is considered as chemotherapy and other non-immunologic novel drugs, ‘Biological’ which refers to immunotherapy, ‘Radiation’ in reference to radiotherapy, ‘Procedural’ that indicates surgical therapy and ‘Diagnostic’ if the trial was studying a diagnostic tool for HPV-related HNSCC. The term ‘Combination’ refers to any trial combining two of the five aforementioned intervention categories while ‘Others’ include interventions not mentioned above. These include medical chart review/patient observation, obtaining human tissue, blood or saliva, performing CHEMRAD assay, Grass SD9 stimulator, detection of tumour DNA, laboratory biomarker analysis.

### Retrieving publications

To determine whether a publication is linked to a clinical trial, the NCT ID associated with each trial was inserted into the PubMed search engine. If any published manuscript were to be linked to the clinical trial, then the respective NCT number was found in the research paper. All articles obtained in the search method were considered in the study, whether they reported trial results/primary outcomes or not.

## Results

### General characteristics

**Figure 2.**
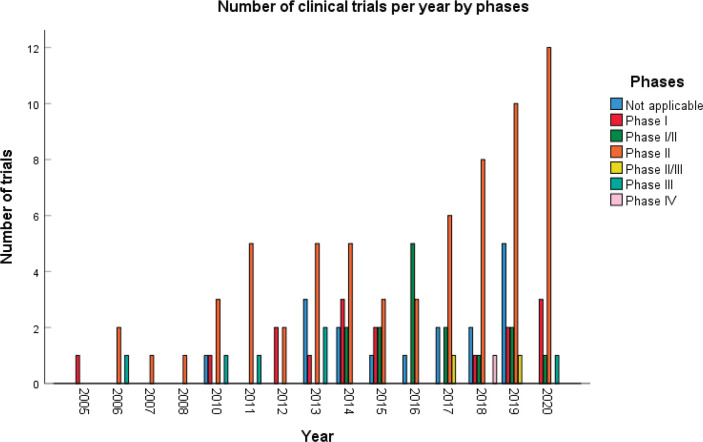
Distribution of clinical trials per year.

A total of 123 trials met the criteria of our study. Table 2 shows the distribution of clinical trial characteristics by phase: the majority of the trials were in phase II (*n* = 66; 53.7%). North American countries (USA and Canada) conducted more than two-thirds of the trials (72.4%) compared to European countries and the rest of the world. Regarding their status, trials were mainly distributed between three categories: recruiting (35.7%), completed (25.2%) or active but not recruiting (22.7%). The lack of publications stemming from 101 clinical trials could be attributed to the fact that their results are still pending. Most of the studies enroled only small amounts of subjects (ranging between 1 and 50 subjects). However, a total of 11,853 individuals were involved across all phases: this is because phases II/III and III recruit a large number of patients with a mean of 593 and 501 subjects, respectively. The average duration of trials ranged between 2 and 5 years with the mean fluctuating around 5 years: this highlights the long amount of time trials need to potentially yield results. Figure 2 shows the pattern of trials conducted per year by phase: trials in a non-applicable phase reached a peak in 2019, while those in phase I, in 2014 and a nadir in 2016–17, the year phases I/II achieved their peak; finally, phase II trials peaked in years 2014 and 2020.

### Not applicable

**Figure 3.**
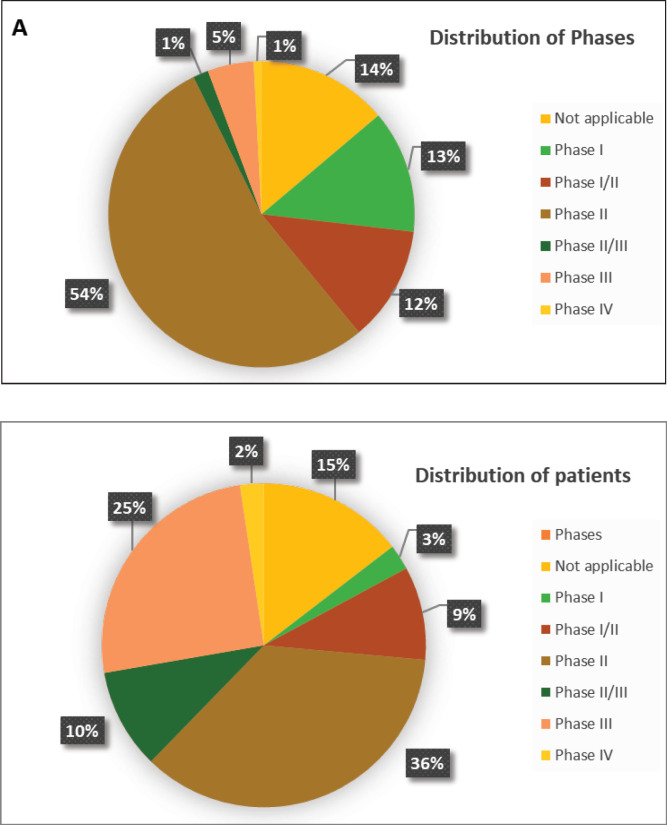
(a and b): Distribution of patients and trials among phases.

**Figure 4.**
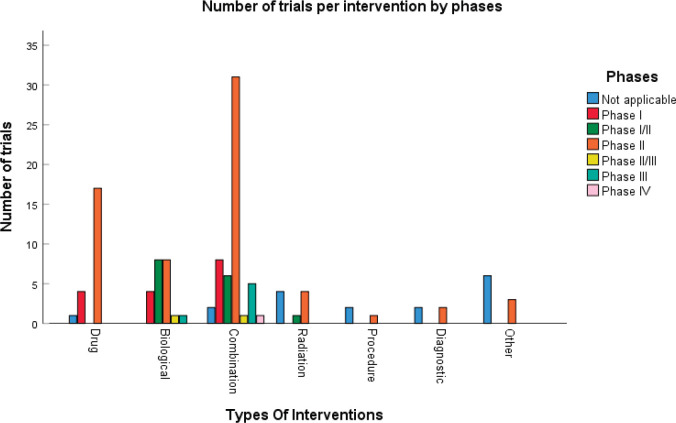
Type of intervention used per clinical trial.

According to Figure 3a and Table 2, 17 (14%) trials were in this phase with only 4 (23.5%) studies being completed while the majority were still recruiting (41.2%). A total of 1,728 patients (15%) were enroled in non-applicable phase trials as evidenced by Figure 3b. The trials were scattered across six different countries with the USA conducting the highest number (10 or 58.8%). The average trial duration in this phase was 5 years. Among the types of interventions listed, trials focusing on radiation therapy for HNSCC were slightly more common than others with 23.5%. However, 35.3% of the trials tackled other topics different from the aforementioned ones (Figure 4). Primary endpoints were evenly distributed between trials with the detection technique being the most common primary endpoint with a total of three (Table 1).

### Phase I

Phase I trials constitute 13% (*n* = 16) of all trials included in this study and only 3% of patients were enroled in this phase (*n* = 301) as evidenced by Figure 3, panels a and b, respectively. Only the United States (13), Germany (2) and the Netherlands (1) performed phase I trials. Most of these trials were completed (35.3%) but all showed no results (Table 2). The average trial duration was 3.4 years. As seen in Figure 4, trials were divided equally between those focusing on immunotherapy as a treatment for HNSCC and those studying the effect of chemotherapy and another novel drug. Eight studies (50%) combined their interest in both types of treatment as well as radiation or procedural therapy. The primary endpoint among phase I trials was dose-limiting toxicities (DLTs) (35.3%) followed by adverse events and maximally tolerated dose (17.6%) (Table 1).

### Phase I/II

Phase I/II trials represent 12.2% of all included trials (*n* = 15), the majority of which (*n* = 10) were held in the United States with an active status but not recruiting. Only two trials were completed and consequently, two trials yielded results. The average trial duration was 5.13 years (Table 2). Interventions used in this phase were restricted to two main treatments: biological (*n* = 8) and combination therapy (*n* = 6) with only one study using radiation (Figure 4). The primary endpoint of trials in phase I/II was divided mainly into the study of adverse events (*n* = 4; 26.7%) and the study of response rate (*n* = 4; 26.7%) (Table 1).

### Phase II

Trials in phase II constituted more than half of those included in this study (53.7%) enroling 36% of the total patients (Figure 3). Similar to phase I/II trials and all other phases, the United States conducted the most trials (72.7%) (Table 2). 40.9% (*n* = 27) of trials in phase II were in the state of recruitment while only 25.7% were completed (*n* = 17) showing results. The average trial duration was 4.44 years. Of these trials and others done throughout the years, interventions were wide but the most widely used was drug combination (41.9%; *n* = 31) (Figure 4). Consequently, 28.8% (*n* = 19) studied response rate and 21.2% (*n* = 14) studied progression-free survival among others, but no phase II trials studied maximum tolerated dose, quality of life (QOL), detection techniques, DLT and disease-free survival (DFS) (Table 1).

### Phase II/III, III, IV

Phases II/III, III and IV represent 1%, 5% and 1% of trials included in this study, respectively, with the involvement of 10%, 25% and 2% of patients (Figure 3). One phase II/III trial was suspended while one phase III trial was terminated but two were completed ending in results. These two phases have a similar average duration of 6.5 years. The only phase IV trial included was conducted in Germany in 2018 and is still active but not recruiting (Table 2). Combination therapy was again the most used intervention in these phases (Figure 4). The primary outcome of phase III trials was overall survival (OS) (*n* = 3) followed by DFS (*n* = 2) (Table 1).

### Publications linked to trials

From the 123 trials included in this study, only 30 had their NCT identification number linked to publications. Despite having the overall highest number of publications (*n* = 16), only 24.2% of phase II trials had linked publications. In contrast, five out of six phase III trials ended up in publications (83.3%) and only 41.9% of completed trials were published. Active but not recruiting trials were slightly more successful: half of them resulted in publications at the end of the trial. Withdrawn, terminated and suspended trials produced only one publication, all three combined. Not all publications linked to trials are related to the primary results of the clinical trial: less than half (46.7%) of the publications stemmed from trials with results.

## Discussion

This study identified 123 trials pertaining to HPV-related HNSCC, of which most were phase II trials (53.7%) that also yielded the highest number of publications (16). Overall, 30 publications resulted from the 123 trials and only half of the published papers (46.7%) exposed primary results. As a matter of fact, 101 trials had no results to date and over the last 15 years, there is not a single phase I study that yielded results. Most phase III trials gave way to publications (five out of six) whereas only one publication ensued from the 20 trials that were either withdrawn, terminated or suspended. Most trials emanated from North America, involved between 0 and 50 participants, and lasted between 2 and 5 years. Overall the most commonly investigated intervention was a combination of many modalities and the most studied outcome was response rate. However, the primary outcome of phase III trials was OS followed by DFS.

Our study was able to retrieve 123 trials with 30 publications happening over the last 15 years. This number can be considered fair when compared to numbers found in other studies. For example, a similar study conducted on clinical trials for hypertrophic cardiomyopathy (HCM) found only 63 therapeutic and interventional trials from the discovery of HCM in 1957 until January 2019 [5]. The authors attributed this low number to the low prevalence of HCM and the heterogeneous pathophysiology of the disease. Conversely, a study reporting the number of clinical trials conducted on another cancer, glioblastoma, found 417 trials in 12 years (from January 2005 until December 2016) and 52 published trials. Nonetheless, and despite all these efforts, only one out of eight completed phase III trials reported positive results, and only 8%–11% of patients with newly diagnosed glioblastoma were enroled in clinical trials [6].

It is evident in our results that the dominant majority of clinical trials were conducted in North America (72.4%). This could be attributed to the high prevalence of this cancer type in such a geographic region when compared to the rest of the world [7]. Nonetheless, countries within this region are considered developed countries and thus possess the necessary means to conduct these trials, whether in terms of resources or infrastructure [8]. Such high-income countries are also well equipped for cancer diagnosis and registration. This is further validated by the fact that only the United States, Germany and the Netherlands performed phase I trials. In contrast, less income countries did not contribute to the same extent for this particular subject. Possible reasons include lack of funding and possible stability issues in developing regions.

The distribution of patients over phases mainly clusters around phases II and III representing 36% and 25% of the total, respectively (Figure 3b). However, when reflecting on the distribution of trials, the preceding dominance stands only for phase I at 54% of the total and abates for phase III at 5% of the total (Figure 3a). This can be explained by the nature of phases of clinical trials, as they tend to recruit larger numbers of patients as they progress sequentially from phase I through IV. In concordance, our study shows that all six trials in phase III enrol more than 100 patients and the great majority of trials in phase II enrol between 0 and 50 patients per trial (Table 2). Moreover, a great sum of trials fails in phase II, and even those that complete phase II are poor predictors of the completion of phase III. That being said and the fact that phase III is the most expensive and time-consuming could explain the decline in trials in said phase as it carries the highest stakes at risk [10].

Scientific knowledge is cumulative by nature and relies on a process of trial and error to capture findings. Here comes the importance of recording these findings in terms of publications to inspire studies of better design and allow researchers to avoid errors of prior colleagues in retrospect. However, our study showed that only 1 of 20 trials conducted that were withdrawn, terminated or suspended yielded publication and merely 41.9% of those completed did so. This exposes future researchers to a higher chance of trial failure and wastes valuable resources in terms of time, personnel and funding. Ultimately, this culminates in hindering the efficiency of scientific progression. Nonetheless, the fault may not rely simply on the investigators themselves as journals tend to favour publications with positive results [11]. This introduces bias to meta-analysis and consequently misrepresents factual information to decision-makers, whether they are patients, physicians or policymakers.

When looking at the primary outcome of trials by phase, it is not a surprise that it is specific to the general purpose of the phase in question. As for the resulting output, none of the phase I trials gave way to results. In fact, Phase I trials aim to assess the safety of the treatment: a small number of healthy people are given the smallest dose and then monitored for the occurrence of adverse events to progressively increment the dose and reach the highest tolerated dose. For this reason, many phase I trials fail as a consequence of adverse events experienced. Another reason for the lack of results may be financial where studies fail to reach an outcome due to lack of funding [12].

Our study looks solely into HPV-positive HNSCC. HNSCC trends have been decreasing in the past few years, which has been attributed to decreased tobacco use [13]. In contrast, cases of oropharyngeal cancer associated with HPV have been increasing [14]. This observation is particularly important as it was found that patients with HPV-positive oropharyngeal cancer have a more favourable prognosis [15, 16]. Thus, testing for HPV status became an inherent part of oropharyngeal cancer staging. As a result, in 2017, a new staging system was put together by the American Joint Commission on Cancer (AJCC) [17, 18] and the Union for International Cancer Control (UICC) which differentiates stratification for patients with HPV-positive HNSCC and HPV-negative HNSCC (TNM classification 8th edition issued by the AJCC and the UICC). In this new classification, staging for HPV-negative oropharyngeal cancer goes from stage 0 to stage IVC with a gradually worsening prognosis for stages IVA, IVB and IVC, whereas classification for HPV-positive oropharyngeal cancer goes from stage 0 to only stage IV without sub-classifications. Despite the aforementioned new classification, current data is not robust enough to recommend a different less intensive treatment regimen for HPV-positive cancer [19, 20]. Over-treating a less aggressive cancer is of special concern because patients usually have a longer life expectancy and are left with the toxic sequelae of an intensive treatment course which impairs their QOL. In this context, more efforts need to be employed to implement new guidelines for patients with HNSCC who test positive for HPV.

Concerning therapeutic modalities, many questions also remain unanswered and could serve as objectives for future trials. For instance, most cases of HNSCC present in late stages with either extensive local invasion or metastasis. For these patients, primary chemoradiotherapy is sometimes the treatment of choice, especially when surgery is not technically feasible. The recommended chemotherapy regimen to accompany radiotherapy is high-dose cisplatin. Cisplatin is preferably used in non-elderly patients with relatively good health due to its side effects profile [22]. This alkylating agent is known to cause nephrotoxicity in up to 35% of cases, myelosuppression in 25%–30% of patients and possibly hepatotoxicity and neuropathy. For patients with multiple co-morbidities requiring chemoradiotherapy, current trials should focus on finding alternative chemotherapy regimens with more acceptable side effects. As for using induction chemotherapy with taxane prior to chemoradiotherapy, available data from the performed studies are conflicting, and more trials are also needed [23, 24].

Other major points about HNSCC need to be elucidated which requires the deployment of more clinical trials with different objectives. As the majority of trials aimed at testing the effects of therapeutic regimens for HNSCC (mostly combinations of different treatment modalities), other trials are needed to evaluate the effectiveness of HPV prophylactic vaccination against oropharyngeal cancer, as this vaccination is recommended to avoid HPV-related cervical and anogenital cancer [22].

## Limitations

One limitation of this study is the exclusion of trials on HPV-negative HNSCC and focusing only on HPV-positive disease. Future studies would be needed to review trials on the former and possibly compare the results between the two.

## Conclusion

Over the last 15 years, 123 clinical trials concerning HPV-positive HNSCC were executed, the majority of which were phase II trials also providing the highest output of publications; nonetheless, publications are still scarce relative to the number of trials. Despite falling in the middle of the spectrum with respect to the number of trials when compared to landscapes studying other diseases, our research highlights the need for even more trials tackling multiple aspects of HPV-positive HNSCC: treatment modalities for less aggressive cancer types and alternative chemoradiotherapy regimens with possibly fewer side effects in patients suffering from several co-morbidities. There is also a need for publications from such trials to build on previous drawbacks as well as successes.

## Conflicts of interest

The authors declare that they have no conflicts of interest.

## Authors’ contributions

Yara Bteich and Jad Hosri contributed equally through data collection, statistical analysis and manuscript writing. Jad Wehbi and Lea Daou also contributed to manuscript writing.

## Funding

## Figures and Tables

**Table 1.** Primary outcomes of clinical trials by phases.

	NA	Phase I	Phase I/II	Phase II	Phase II/III	Phase III	Phase IV	Total
Primary outcome								
Adverse events	1	3	4	5	0	0	0	13
MTD	0	3	1	0	0	0	0	4
Local recurrence	1	0	0	1	0	0	0	2
Quantitative assessment of dPET	1	0	0	1	0	0	0	2
Clearance of HPV biomarkers	1	0	0	1	0	0	0	2
Percent weight loss	0	0	0	1	0	0	0	1
EFS	0	0	0	2	0	0	0	2
QOL	1	0	0	0	0	0	0	1
Detection technique	3	0	0	0	0	0	0	3
Other	3	0	0	4	0	1	1	9
Response rate	0	0	4	19	0	0	0	23
PFS	0	0	1	14	1	0	0	16
DLT	0	6	2	0	0	0	0	8
OS	1	0	0	3	0	3	0	7
DFS	2	0	0	0	0	2	0	4
Change in marker level	2	2	0	5	0	0	0	9
Loco-regional control	1	0	0	4	0	0	0	5
Several	0	2	3	6	1	0	0	12

MTD, Maximum tolerated dose; dPET, Dynamic positron emission tomography; EFS, Event-free survival; QOL, Quality of life; PFS, Progression-free survival; DLT, Dose-limiting toxicity; OS, Overall survival; DFS, Disease-free survival

**Table 2.** Characteristics of clinical trials on HPV-positive HNSCC as found in ClinicalTrials.gov (2005–2020).

	NA	Phase I	Phase I/II	Phase II	Phase II/III	Phase III	Phase IV	Total
Trials								
Number of trials	17 (13.8%)	16 (13%)	15 (12.2%)	66 (53.7%)	2 (1.6%)	6 (4.9%)	1 (0.8%)	123 (100%)
Location								
United States	10	13	10	48	2	3	0	86 (69.9%)
France	2	0	0	8	0	0	0	10 (8.13%)
Germany	1	2	0	1	0	1	1	6 (4.88%)
United Kingdom	0	0	1	1	0	0	0	2 (1.6%)
The Netherlands	0	1	1	1	0	0	0	3 (2.44%)
Canada	1	0	0	2	0	0	0	3 (2.44%)
Denmark	2	0	0	1	0	0	0	3 (2.44%)
Others	1	0	3	4	0	2	0	10 (8.13%)
Status								
Terminated	2	2	0	6	0	1	0	11 (8.94%)
Completed	4	6	2	17	0	2	0	31 (25.20%)
Recruiting	7	3	5	27	1	1	0	44 (35.77%)
Active, not recruiting	3	3	7	12	0	2	1	28 (22.76%)
Withdrawn	1	2	1	3	0	0	0	7 (5.69%)
Suspended	0	0	0	1	1	0	0	2 (1.63%)
Study results								
No results	16	16	13	49	2	4	1	101 (82.1%)
Has results	1	0	2	17	0	2	0	22 (17.9%)
Enrolment								
0–50	8	15	8	39	0	0	0	70 (56.9%)
51–100	4	1	5	13	0	0	0	23 (18.7%)
>100	5	0	2	14	2	6	1	30 (24.4%)
Study duration								
0–1	4	5	0	10	0	0	0	19 (15.44%)
2–5	6	7	8	35	1	2	1	60 (48.78%)
6–9	4	4	6	19	1	3	0	37 (30.08%)
>10	3	0	1	2	0	1	0	7 (5.69%)
